# Novel Antioxidant Peptides Identified from *Arthrospira platensis* Hydrolysates Prepared by a Marine Bacterium *Pseudoalteromonas* sp. JS4-1 Extracellular Protease

**DOI:** 10.3390/md21020133

**Published:** 2023-02-20

**Authors:** Congling Liu, Gong Chen, Hailian Rao, Xun Xiao, Yidan Chen, Cuiling Wu, Fei Bian, Hailun He

**Affiliations:** 1 State Key Laboratory of Medical Genetics, School of Life Sciences, Central South University, Changsha 410013, China; 2 Department of Biochemistry, Changzhi Medical College, Changzhi 046000, China; 3 Institute of Crop Germplasm Resources, Shandong Academy of Agricultural Sciences, Jinan 250100, China

**Keywords:** phycobiliprotein, antioxidant peptide, bacterial extracellular protease, enzymatic hydrolyzation, *C. elegans*

## Abstract

Crude enzymes produced by a marine bacterium *Pseudoalteromonas* sp. JS4-1 were used to hydrolyze phycobiliprotein. Enzymatic productions showed good performance on DPPH radical and hydroxyl radical scavenging activities (45.14 ± 0.43% and 65.11 ± 2.64%, respectively), especially small peptides with MWCO <3 kDa. Small peptides were fractioned to four fractions using size-exclusion chromatography and the second fraction (F2) had the highest activity in hydroxyl radical scavenging ability (62.61 ± 5.80%). The fraction F1 and F2 both exhibited good antioxidant activities in oxidative stress models in HUVECs and HaCaT cells. Among them, F2 could upregulate the activities of SOD and GSH-Px and reduce the lipid peroxidation degree to scavenge the ROS to protect *Caenorhabditis elegans* under adversity. Then, 25 peptides total were identified from F2 by LC-MS/MS, and the peptide with the new sequence of INSSDVQGKY as the most significant component was synthetized and the ORAC assay and cellular ROS scavenging assay both illustrated its excellent antioxidant property.

## 1. Introduction

The microalgae *Arthrospira* belongs to the cyanobacteria division, the cyanophycean class, and the Oscillatoriaceae family. *Arthrospira* sp. is a photosynthetic cyanobacterium that has recently grown in value as a nutritional supplement and food additive due to its high protein content of around 60–70% in their biomass and various valuable natural compounds, such as pigments, β-carotenes, polysaccharides and peptides. In addition, *Arthrospira* sp. has a wide range of uses in the pharmaceutical, cosmetic, and nutritional industries. Because of its high protein content and nutritive value of amino acid composition, *Arthrospira* sp. was evaluated as a potential meat substitute and has received increased attention [1]. Phycobiliprotein is a class of proteins with antioxidant, antitumor and anti-inflammatory properties, but the heat sensitivity and allergenicity limit its wider applications. Phycobiliprotein is highly abundant in *Arthrospira*, accounting for about 20% of its dry weight [2]. Using proteases to hydrolyze the phycobiliprotein to produce antioxidant peptides is an efficient way to reach higher bioactive potential and promote high value utilization of marine proteins while avoiding its natural limitations. Moreover, the proteins of microalgae can be converted by enzymatic hydrolysis into value-added products with better functional properties such as antioxidant peptides (APs), anti-hypertension peptides, and antibacterial peptides, etc. Therefore, more and more researchers are interested in obtaining effective bioactive peptides (BPs) from *Arthrospira* sp. hydrolysates.

Enzymatic hydrolysis of the dietary proteins generates BPs, which are short chains of 2–15 amino acids residues, can enhance nutritional value, safety, bioactive function and reduce the allergenicity [3,4]. Numerous studies have demonstrated that BPs with antioxidant activity produced by proteolysis can achieve antioxidation by scavenging free radicals, chelating transition metals, and enhancing the activity of endogenous antioxidant enzymes such as catalase (CAT), superoxide dismutase (SOD), and glutathione peroxidase (GSH-Px). Redox homeostasis (balance) is an important cellular process that plays a vital role in maintaining the normal physiological steady state of the human body. A disturbance of balance between oxidants and antioxidants results in oxidative stress. Reactive oxygen species (ROS) are molecules derived from aerobic cellular metabolism; in the oxidative stress condition excessive production of ROS can disrupt the intracellular redox balance and cause numerous diseases such as cancer, diabetes, atherosclerosis, cardiovascular and neurodegenerative [4]. A growing number of studies have demonstrated that APs from protease hydrolysates can effectively scavenge free radicals or inhibit the production of ROS. Meanwhile, food-derived APs also performed nontoxicity, are low cost, absorb efficiency advantages, and were considered as potential substitutes for commercial synthetic antioxidants [5,6].

Since different proteases have specific hydrolyzed performance on protein substrate and can produce diverse BPs with different length, sequence, and composition, these hydrolyzes may exhibit various hydrophobicity and functionalities [7,8]. Therefore, it is necessary to find a new enzymes resource to hydrolyze *Arthrospira platensis* (*A. platensis*) and produce novel APs with high antioxidant activity and bioactive properties. Our previous study suggested using extracellular proteases secreted by a marine bacterium *Pseudoalteromonas* sp. JS4-1 to hydrolyze *chlorella* can obtain hydrolysates with high antioxidant activity [9]. In this study, proteases from *Pseudoalteromonas* sp. JS4-1 were used to hydrolyze *A. platensis* proteins and produced diverse Aps; a series of biochemical assays, cell models, and animal models were applied for testing the bioactivity of the APs.

## 2. Results

### 2.1. Screening of Proteases for Enzymatic Hydrolysis

**Figure 1:**
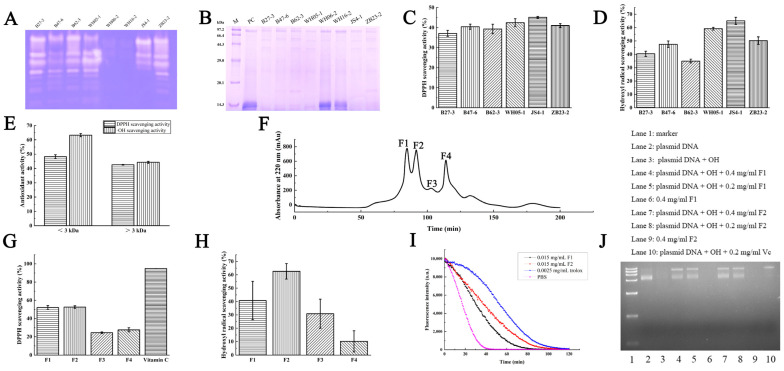
Antioxidant activities of phycobiliprotein hydrolysis products with JS4-1. (**A**) Comparison of catalytic ability on casein of proteases from *Pseudoalteromonas* sp. B27-3, *Pseudoalteromonas* sp. B47-6, *Pseudoalteromonas* sp. B62-3, *Pseudoalteromonas* sp. WH05-1, *Pseudoalteromonas* sp. WH06-2, *Pseudoalteromonas* sp. WH16-2, *Pseudoalteromonas* sp. JS4-1 and *Pseudoalteromonas* sp. ZB23-2. (**B**) SDS-PAGE electrophoresis of phycobiliprotein and hydrolysates of different proteases (Line M: protein marker, Line PC: phycobiliprotein). (**C**) DPPH radical and (**D**) hydroxyl radical scavenging activity of phycobiliprotein hydrolysates of different proteases. (**E**) Antioxidant activities of the ultrafiltration fraction of phycobiliprotein hydrolysis products treated with optimized hydrolysis condition. Data were expressed as mean ± SD (*n* = 3). (**F**) Size-exclusion liquid chromatography of the <3 kDa ultrafiltration fractions on a Sephadex LH-20 column. The F1–F4 represent purified fractions. (**G**) DPPH radical scavenging activity of F1-F4 compared with Vitamin C. (**H**) Hydroxyl radical scavenging activity of F1–F4. (**I**) Peroxyl radical scavenging activity (ORAC) of F1 and F2 compared with PBS and Trolox. (**J**) Protective effects of F1 and F2 on hydroxyl radical-induced oxidation of plasmid DNA compared with Vitamin C. Data were expressed as mean ± SD (*n* = 3).

*Pseudoalteromonas* is a strictly marine genus and able to produce extracellular enzymes, which have good advantages in degrading natural marine proteins [10]. In order to evaluate which enzyme is more suitable for hydrolyzing *Arthrospira* sp. proteins, the extracellular enzymes from eight strains named *Pseudoalteromonas* sp. B27-3, *Pseudoalteromonas* sp. B47-6, *Pseudoalteromonas* sp. B62-3, *Pseudoalteromonas* sp. WH05-1, *Pseudoalteromonas* sp. WH06-2, *Pseudoalteromonas* sp. WH16-2, *Pseudoalteromonas* sp. JS4-1 and *Pseudoalteromonas* sp. ZB23-2 were extracted and screened by zymography. The enzymatic hydrolysates were identified by SDS-PAGE and their DPPH RSA and OH RSA activities were also detected. The zymography showed with the exception of *Pseudoalteromonas* sp. WH06-2 and *Pseudoalteromonas* sp. WH16-2, all the other strains can secrete a variety of proteases, indicating that most marine bacteria could produce extracellular proteases with a variety of cleavage sites, and suitable for enzymatic hydrolysis (Figure 1A). Proteases from *Pseudoalteromonas* sp. B27-3 and *Pseudoalteromonas* sp. JS4-1 have stronger hydrolysis activity than other bacterial proteases toward *Arthrospira* sp. proteins. SDS-PAGE showed that the hydrolysates existed in smaller fragments compared with the control protein, and almost no protein band left on the gel, while the hydrolysates produced by other bacterial proteases still left visible bands on the gel (Figure 1B). The hydrolysates of *Pseudoalteromonas* sp. JS4-1 proteases have the highest DPPH RSA (45.14 ± 0.43%) and OH RSA (65.11 ± 2.64%) than other productions (Figure 1C,D). Therefore, proteases from *Pseudoalteromonas* sp. JS4-1 were selected as a candidate for *Arthrospira* sp. proteins hydrolyzing. 

### 2.2. Optimization of Enzymatic Hydrolyzing Parameters

Enzymatic hydrolyzation was optimized using the one-variable-at-a-time (OVAT) approach by changing one parameter while keeping other parameters constant. First, the hydrolyzing was performed at 50 °C from 1 h to 7 h with 1 h at intervals, at a E: S ratio of 1:10, to estimate the optimal hydrolyzing time. Data showed that the hydrolysis degree increased along with the hydrolyzing process, especially in the initial three hours. After five hours hydrolyzation, the hydrolysis degree was not improved significantly. DPPH RSA as an evaluation index suggested that when the E/S ratio ranged from 1:6 to 1:10, the DPPH RSA of the hydrolytes exhibited no significant difference. While the E/S ratio adjusted to 1:12 and 1:14, the DPPH RSA of the hydrolytes dropped 6.72% and 15.95%, respectively, indicating that the substrate exceeded the catalytic capacity of the enzymes. When the hydrolyzing temperature increased, the DPPH RSA improved and reached the maximal activity at 50 °C, but when the temperature exceeded >50 °C the DPPH RSA decreased, probably due to the enzymes being inactivated at higher temperatures. After OVAT optimization, when E:S ratio was 1:10 and enzymatic hydrolyzing performed at 50 °C for 4 h, the DPPH RSA and the OH RSA of the hydrolysates achieved the maximal level of 48.51 ± 2.42% and 67.16 ± 3.21%, respectively. The ninhydrin colorimetry was used to determine the hydrolysis degree, and data showed that about 0.67 ± 0.02 mM of •NH_2_ was released after hydrolyzing reaction.

In order to evaluate the free RSA of small peptides, the hydrolysates were separated into 2 parts by ultrafiltration. The DPPH RSA of < 3 kDa and > 3 kDa parts were 48.26 ± 1.34% and 42.60 ± 0.25%, respectively. The OH RSA of < 3 kDa and > 3 kDa parts were 63.30 ± 1.11% and 44.30 ± 0.68%, respectively (Figure 1E). It has been proven that the small peptides (<3 kDa) contained more antioxidant components and were more easy to exert biological effects [11]. The bioactive peptides with small molecular weight have broad application potential in food, medicine, cosmetics and other industries due to their good absorbability and fewer side effects besides their higher bioactivity [12].

### 2.3. Antioxidant Activities of Size-Exclusion Liquid Chromatography Fractions of Phycobiliprotein Hydrolysis Products

In order to investigate the antioxidant components of the small peptides, the peptides with MWCO <3 kDa were further separated using size-exclusion liquid chromatography, and in total four fractions defined as F1 to F4 were obtained (Figure 1F). By measuring the DPPH RSA (F1 to F4 were 52.06 ± 2.13%, 52.56 ± 1.38%, 24.43 ± 0.85% and 27.58 ± 2.24%, respectively; Figure 1G) and the OH RSA (F1 to F4 were 40.75 ± 14.35%, 62.61 ± 5.80%, 30.86 ± 10.90%, and 10.21 ± 7.91%, respectively; Figure 1H), F1 and F2 showed higher antioxidant activity than F3 and F4. We could obtain about 40 mg of F1 and 40 mg of F2 lyophilized powder after the size-exclusion liquid chromatography from 1 g of *A. Platensis* powder.

Then, the ORAC assay was performed to compare the antioxidant activity of F1 and F2. At peptide concentration of 0.015 mg/mL, both F1 and F2 could significantly slow down the decrease rate of the fluorescence decay compared with PBS. The Trolox equivalent antioxidant capacity of F1 and F2 were 0.27 ± 0.02 mmol TE/g and 0.41 ± 0.04 mmol TE/g, respectively (Figure 1I). F2 exhibited higher antioxidant activity than F1.

The hydroxyl radicals could open the circular of supercoiled DNA (SC DNA) and make it become partly opened circular DNA (OC DNA), which can slow down the DNA mobility on agarose gel. Comparing with the no damaged plasmid DNA pET-22b (Figure 1J, lane 2), when the plasmid DNA has no protection, it can be completely degraded by hydroxyl radicals into small fragments and no band observed on the gel (Figure 1J, lane 3). An amount of 0.2 mg/mL of Vitamin C can protect the plasmid DNA from oxidative damage, but the protection is limited and the OC DNA is the major form retained on the gel (Figure 1J, lane 10). F1 and F2 concentrations range from 0.2 mg/mL to 0.4 mg/mL; all can significantly protect plasmid DNA from oxidative damage and these protections were far better than Vitamin C, which make the SC DNA and OC DNA co-existing on the gel (Figure 1J, lane 4, 5, 7, 8). So, this study proved that both F1 and F2 have strong antioxidant activity to protect DNA from oxidative stress.

### 2.4. Antioxidant Activities of F1 and F2 at Cellular Levels

The intracellular oxidized cell model is a common method that is used to assess the antioxidative capacity of compounds. Vascular endothelial cell injury is associated with several factors, among them oxidative stress is an important cause of cardiovascular disease. APs are able to protect vascular endothelial cell function which is a key point in the prevention and treatment for cardiovascular diseases. The skin is the first immune defense of the body and susceptible to a variety of physical and chemical stimuli. Protecting skin cells could be an effective strategy to prevent and treat dermatosis. Therefore, we chose HUVECs and HaCaT cells as cellular models to investigate the intracellular antioxidant effects of F1 and F2 [13,14,15,16].

**Figure 2:**
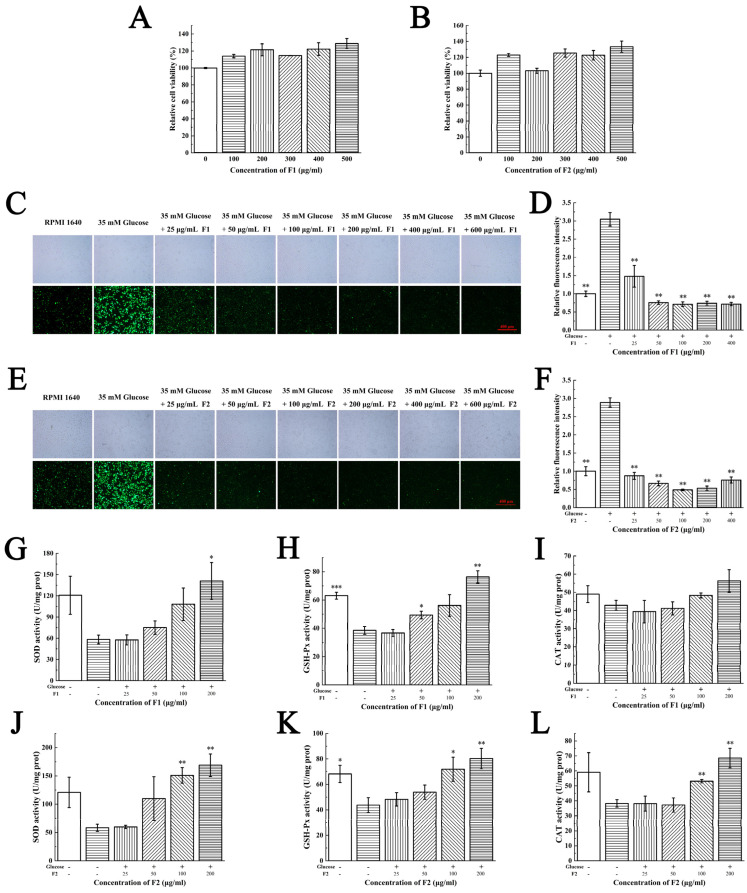
Antioxidant activities of F1 and F2 on HUVEC cells. The effects of different concentration of (**A**) F1 and (**B**) F2 on cell viability were measured by MTT assay. ROS scavenging capacities of (**C**) F1 and (**E**) F2 on HUVEC cells indicated as a green DCFH-DA fluorescence. Images were taken using fluorescence microscope (magnification, 10×). (**D**,**F**) Statistical analysis was performed to quantify relative fluorescence density accordingly (Student *t*-test). (**G**,**J**) Superoxide dismutase (SOD), (**H**,**K**) glutathione peroxidase (GSH-Px) and (**I**,**L**) catalase (CAT) activities in HUVEC cells were detected after treatment with F1 and F2, respectively. The Normality of data was analyzed by Shapiro–Wilk test. Data were expressed as mean ± SD (*n* = 3). * represents *p* < 0.05, ** represents *p* < 0.01 and *** represents *p* < 0.001, compared with glucose group (Student *t*-test).

**Figure 3:**
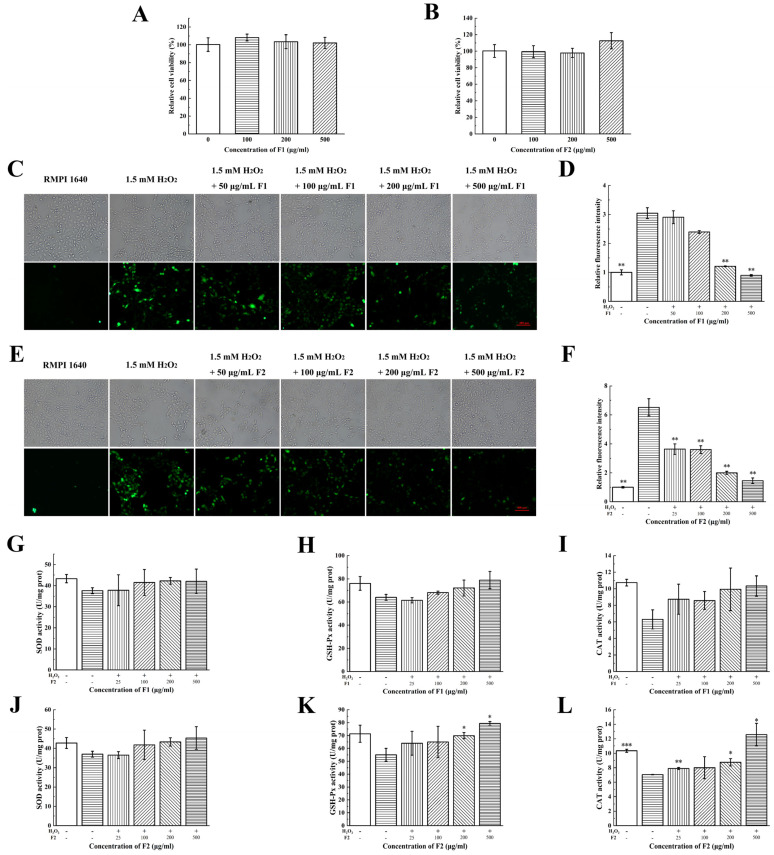
Antioxidant activities of F1 and F2 on HaCaT cells. The effects of different concentration of (**A**) F1 and (**B**) F2 on cell viability were measured by MTT assay. ROS scavenging capacities of (**C**) F1 and (**E**) F2 on HaCaT cells indicated as green DCFH-DA fluorescence. Images were taken using fluorescence microscope (magnification, 10×). (**D**,**F**) Statistical analysis was performed to quantify relative fluorescence density accordingly (Student *t*-test). (**G**,**J**) Superoxide dismutase (SOD), (**H**,**K**) glutathione peroxidase (GSH-Px) and (**I**,**L**) catalase (CAT) activities in HaCaT cells were detected after treatment with F1 and F2, respectively. The Normality of data was analyzed by Shapiro–Wilk test. Data were expressed as mean ± SD (*n* = 3). * represents *p* < 0.05, ** represents *p* < 0.01 and *** represents *p* < 0.001, compared with H_2_O_2_ group (**G**,**J** Mann–Whitney test; **H**,**I**,**K**,**L** Student *t*-test).

MTT assay indicated that both F1 and F2 had no significant cytotoxicity on HUVECs and HaCaT cells; moreover, they can obviously increase the cell viability at a concentration range of 100–500 μg/mL (Figure 2A,B and Figure 3A,B). 

An amount of 35 mM of glucose was added to the HUVECs and 1.5 mM H_2_O_2_ was used in the HaCaT cells. Cells treated with glucose and H_2_O_2_ exhibited higher fluorescence intensity than control groups, indicating the oxidative stress models have been successfully established (Figure 2C,E and Figure 3C,E). The intracellular ROS were labelled by DCFH-DA probe. Cells treated with low concentration of F1 and F2 (25 μg/mL) displayed less fluorescence intensity than the glucose group in HUVECs, suggesting the fractions were able to scavenge intracellular ROS to protect HUVECs from hyperglycemia-induced oxidative stress (Figure 2D,F). Cells treated with a high concentration of F1 (100 μg/mL) and a low concentration of F2 (50 μg/mL) displayed less fluorescence intensity than the H_2_O_2_ group in HaCaT cells, suggesting that the fractions were able to scavenge intracellular ROS to protect HaCaT cells from H_2_O_2_ induced oxidative stress (Figure 3D,F). The antioxidant activities of peptides on a cellular level in this study were close to those reported extracts of plants [17,18,19], which suggested the peptides in our study may also have great potential applications.

To further investigate the underlying mechanisms of fractions scavenging intracellular ROS, we detected the activities of antioxidant enzymes such as SOD, GSH-Px and CAT after being treated with fractions due to their role in scavenging ROS and suppressing oxidative stress [20,21]. The glucose and H_2_O_2_ reduced the activities of SOD, GSH-Px and CAT in HUVECs and HaCaT cells. The administration of F1 and F2 dose-dependently increased the activities of these enzymes at a concentration range of 25–200 μg/mL (Figure 2G–L and Figure 3G–L).

### 2.5. Effects of F2 on Resistance to Oxidative Stress in C. elegans 

*Caenorhabditis elegans* (*C. elegans*) have many highly conserved genes and signaling pathways involved in the regulation of aging and oxidative stress [22,23]. The genes and pathways in aging, oxidative stress and inflammation between *C. elegans* and human are highly homologous [24]. *C. elegans* as an animal model is widely used for anti-aging and anti-oxidative studies.

**Figure 4:**
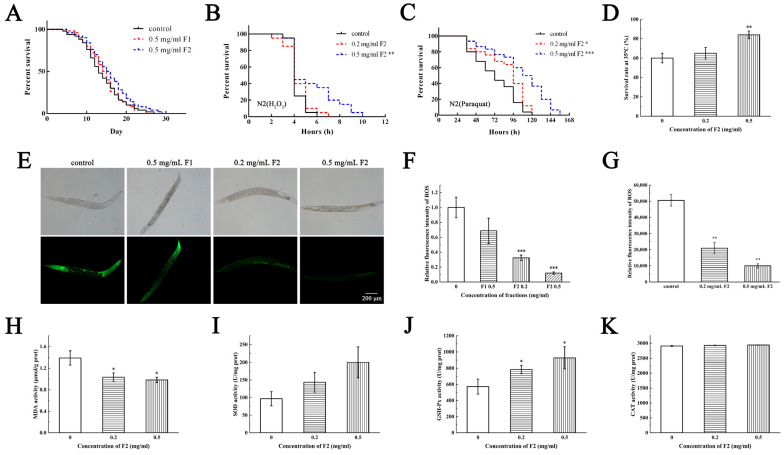
Effects of F2 on resistance to oxidative stress in Caenorhabditis elegans. (**A**) Survival curves of C. elegans treated with F1 and F2. Survival curves of C. elegans pre-treated with F2 at given concentrations for 3 days and then exposed to (**B**) 10 mM H_2_O_2_ and (**C**) 10 mM paraquat. * represents *p* < 0.05, ** represents *p* < 0.01 and *** represents *p* < 0.001, compared with H_2_O_2_ and paraquat group (Student *t*-test). (**D**) Survival rate of C. elegans pre-treated with F2 at given concentrations for 3 days and then cultured at 35 °C for 10 h. (**E**) Antioxidant activities of F2 on *C. elegans* were detected by DCFH-DA probe. Images were taken using fluorescence microscope (magnification, 5×). (**F**) Statistical analysis was performed to quantify relative fluorescence density (Student *t*-test). (**G**) The ROS content in *C. elegans* was determined based on the average integrated area of the fluorescene intensity of probes during 6 h intervals. (**H**) MDA levels, (**I**) superoxide dismutase (SOD), (**J**) glutathione peroxidase (GSH-Px) and (**K**) catalase (CAT) activities in C. elegans were detected after treated with F2 for 3 days. The Normality of data was analyzed by Shapiro–Wilk test. Data were expressed as mean ± SD (*n* = 3). * represents *p* < 0.05, ** represents *p* < 0.01 and *** represents *p* < 0.001, compared with blank control group (Student *t*-test).

In this study, 0.5 mg/mL of F1 and F2 had no toxicity and no significant life-extending effect on N2 (Figure 4A). The in vivo ROS assay showed that 0.5 mg/mL of F1 had no significant ROS scavenging effects, while F2 could scavenge ROS at low concentration (0.2 mg/mL, Figure 4E,F). The fluorescence accumulation assay of 80 nematodes showed similar results (Figure 4G). 

Then, we speculate whether the APs can enhance the stress resistance of N2. Oxidizing agent H_2_O_2_ (10 mM), pesticide paraquat (10 mM) and heat (35 °C) were chosen as stress conditions. Considering F2 has higher anti-oxidative activity than F1, in this study N2 were treated with F2 and then exposed to stress conditions. Data showed that the F2 can extend the stressed N2 survival time in all stress conditions, and the survival rate was positively correlated with the addition of F2. An amount of 0.2 mg/mL of F2 increased 35% survival rate after 7 h of H_2_O_2_ stress, 50% after 120 h of paraquat stress and 24% under heat stress (Figure 4B–D). Although F2 did not help the *C. elegans* live longer, it really improved the resistant ability of *C. elegans* obviously to the oxidation, pesticide and heat stress.

We then investigated the MDA levels and activities of antioxidant enzymes in nematodes. The MDA level is an important biomarker of lipid peroxidation [25]. F2 could significantly reduce the MDA levels in nematodes (Figure 4H). As shown in Figure 4I–K, F2 could have dose-dependently increased the activities of SOD and GSH-Px, while it had no effects on CAT. These results suggested that F2 could upregulate the activities of SOD and GSH-Px and reduce the lipid peroxidation degree to scavenge the ROS in the organisms. Activating these antioxidant enzymes contributes to maintaining the redox balance, delaying aging process and prolonging lifetime [26]. Antioxidant enzymes with high activity are able to reduce ROS levels, maintain normal physiological steady state and prevent numerous diseases such as cancer, diabetes, neurodegenerative diseases and so on [4]. 

### 2.6. Antioxidant Activities of Synthesized Peptide INSSDVQGKY

Since F2 has the highest antioxidant activity in all fractions, its components were investigated using LC-MS/MS. The total ion chromatography of F2 was shown in Figure S1. A total of 25 peptides with relative abundance >1% were identified, and their sequence and abundance were listed in Table 1. Among them, one peptide with the sequence of INSSDVQGKY accounting for 17.83% was considered as the major component of F2. The MS/MS of INSSDVQGKY was shown in Figure S2.

**Figure 5:**
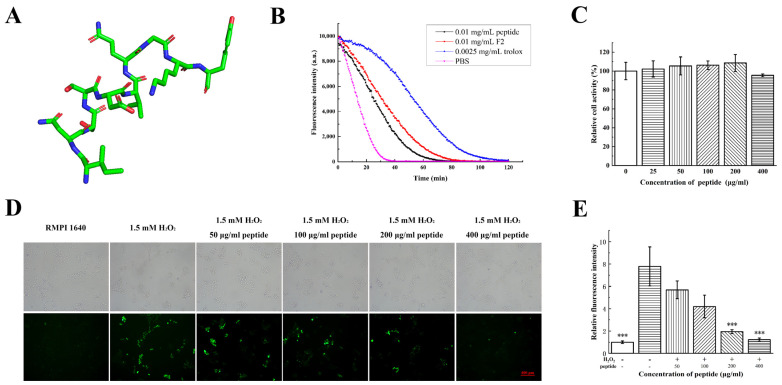
Antioxidant activities of synthesized peptide INSSDVQGKY. (**A**) Predicted structure of peptide INSSDVQGKY. (**B**) ORAC assay of synthesized peptide INSSDVQGKY compared with Trolox and PBS. (**C**) The effects of different concentration of synthesized peptide INSSDVQGKY on HaCaT cell viability were measured by MTT assay. (**D**) Antioxidant activities of synthesized peptide INSSDVQGKY on HaCaT cells were detected by DCFH-DA probe. Images were taken using fluorescence microscope (magnification, 10×). (**E**) Statistical analysis was performed to quantify relative fluorescence density. The Normality of data was analyzed by Shapiro–Wilk test. Data were expressed as mean ± SD (*n* = 3). *** represents *p* < 0.001, compared with H_2_O_2_ group (Student *t*-test).

Sequence alignment showed that the peptide INSSDVQGKY was a part of a beta-subunit of allophycocyanin. This was a novel AP first be sequenced and proved with antioxidant activity. The structure of INSSDVQGKY was predicted and it was a linear peptide with a little helix (Figure 5A). In order to validate the antioxidant function of this peptide, it was synthetized. ORAC assay of the synthetized peptide was 0.32 ± 0.03 mmol TE/g and slightly lower than F2 (0.41 ± 0.04 mmol TE/g) (Figure 5B). The peptide had no significant cytotoxicity on HaCaT cells within 400 μg/mL (Figure 5C). The peptide dose-dependently reduced the intracellular ROS levels at a concentration range from 100 μg/mL to 400 μg/mL (Figure 5D,E). This peptide is therefore hypothesized to be the major antioxidant component of F2. The antioxidant activity of INSSDVQGKY is close to peptide PNN which was obtained from hydrolysis of phycobiliprotein by protease K [27] and may have potential applications in further study.

## 3. Discussion

Phycobiliprotein is an ideal marine protein resource with antioxidant properties widely spread in Rhodophyta and Cyanophyta, which could be used in bioactive peptides preparation [28]. This study aimed to apply the bacterial extracellular protease in preparing antioxidant peptides from phycobiliprotein. To utilize phycobiliprotein more comprehensively and avoid its natural limitations, we reported bacterial extracellular protease of *Pseudoalteromonas* sp. JS4–1 hydrolyzed phycobiliprotein to obtain antioxidant peptides in a single process. The bioactive fractions exhibited antioxidant activities both in vitro and in vivo. The biochemical mechanisms of the antioxidant activities of the fractions at cellular and *C. elegans* levels may be through activating the activities of antioxidant enzymes and scavenging the oxidative stress in the organisms. The molecular mechanism underlying this biological progress still need to be further studied in the future.

In this study, extracellular proteases from marine bacteria were used to hydrolyze phycobiliprotein. Marine proteases can degrade organics in the ocean naturally; thus, they have extraordinary advantages in digesting marine-sourced proteins such as fish and plants compared with land proteases [9,29,30]. Compared with commercial proteases, marine proteases have higher catalyzing efficiency and shorter hydrolyzing time. The unique cleavage sites of marine proteases also produced peptides with different amino acid sequences which is helpful to select more bioactive peptides and develop various applications of phycobiliprotein [31]. The identified peptide INSSDVQGKY exhibited antioxidant activities at a concentration of 100–200 μg/mL which was close to some newly found antioxidant peptides derived from other resources [32,33]. The cellular ROS scavenging assay is strong proof of the outstanding antioxidant activity of the peptide during biological process.

ROS are important mediators of biological process. The increased formation of ROS could cause oxidative stress which is associated with oxidative damage to biomolecules [34]. Oxidative stress happens in various pathophysiological processes and is an important pathophysiological mechanism underlying many diseases. Thus, decreasing the ROS levels to inhibit the oxidative stress has become an efficient and promising way in medicine [35]. Activating endogenous antioxidant enzymes such as SOD, CAT and GSH-Px by small molecules is an effective and protective approach in diseases [36]. The enzymatic hydrolysates and newly identified peptide in this study were able to activate the antioxidant enzymes to scavenge ROS under exogenous stress such as high glucose, and H_2_O_2_. Li G et al. identified a novel peptide EDEQKFWGK with DPPH RSA 26.76% and the OH RSA 32.19% from porcine plasma hydrolysate, and this peptide could increase SOD, CAT, and GSH-Px activities in HepG2 cells [37]. Park YR et al. identified a novel peptide NCWPFQGVPLGFQAPP with DPPH RSA 50% from clam worms and exhibited good antioxidant and anti-inflammation effects in RAW264 7 cells [38]. Compared with antioxidant peptides from other resources, phycobiliprotein hydrolysates also showed good antioxidant activities (DPPH RSA: 45.14 ± 0.43% and OH RSA: 65.11 ± 2.64%). Further, phycobiliprotein can be easily obtained and produced massively in the industry. So, exploring bioactive peptides from phycobiliprotein is of great economic value.

Conclusively, the enzymatic hydrolysis of phycobiliprotein is a promising and efficient method to produce antioxidant peptides. The peptides hydrolyzed from phycobiliprotein by marine proteases exhibited antioxidant activities both at cellular levels and in *C. elegans*. 

## 4. Materials and Methods

### 4.1. Strains and Reagents

The *A. platensis* powder was purchased from a local supplier in Shanghai. Strains used in this study were isolated from the sediment of the South China Sea and stored in the lab: *Pseudoalteromonas* sp. JS4-1 (GenBank accession number: MT116988), *Pseudoalteromonas* sp. B27-3, *Pseudoalteromonas* sp. B47-6, *Pseudoalteromonas* sp. B62-3, *Pseudoalteromonas* sp. WH05-1, *Pseudoalteromonas* sp. WH06-2, and *Pseudoalteromonas* sp. ZB23-2. Immortal human umbilical vein endothelial cell line (HUVECs) and human immortal keratinocyte line (HaCaT) were obtained from the National Collection of Authenticated Cell Cultures (Shanghai, China). Wild type *C. elegans* (N2) were obtained from Caenorhabditis Genetics Center (CGC). 

Ninhydrin, penicillin-streptomycin solution (cell culture grade), trypsin-EDTA solution, dimethyl sulfoxide (DMSO), thiazolyl blue tetrazolium bromide (MTT), and phosphate buffered saline (PBS) were purchased from Sangon Biotech (Shanghai, China). Trisodium citrate dihydrate, SnCl_2_, n-propanol, FeSO_4_, 30% H_2_O_2_, cholesterol and glucose were purchased from Sinopharm Chemical Reagent Co., Ltd. (Shanghai, China). In addition, 1,1-diphenyl-2-picrylhydrazyl (DPPH), 1,10-phenanthroline monohydrate (OP), fluorescein sodium salt, 2,20-azobis (2-amidinopropane) dihydrochloride (AAPH), 6-hydroxy-2,5,7,8-tetramethylchroman-2-carboxylic acid (Trolox), L-leucine, antifade mounting medium and NaN_3_ were purchased from Sigma-Aldrich, Ltd. (St. Louis, MO, USA). Amicon^TM^ Ultra-15 centrifugal filter units were purchased from Millipore^®^ (Billerica, MA, USA). Sephadex LH-20 was purchased from GE Healthcare Life Sciences (Uppsala, Sweden). Fetal bovine serum (FBS) was purchased from PAN-BiotechGibco company (Aidenbach, Germany). RPMI-1640 medium was purchased from Gibco^®^ ThermoFisher Scientific Company (Waltham, MA, USA). Methyl viologen dichloride was purchased from Aladdin^®^ (Shanghai, China). BCA assay kit, cell lysis buffer, ROS assay kit, catalase (CAT) assay kit, total superoxide dismutase (SOD) assay kit with NBT, cellular glutathione peroxidase (GSH-Px) assay kit with NADPH and lipid peroxidation MDA assay kit were purchased from Beyotime Biotechnology (Shanghai, China).

### 4.2. Extraction of Phycobiliprotein and Screening of Proteases

#### 4.2.1. Extraction of Phycobiliprotein from *A. platensis*

One gram of *A. platensis* powder was stirred in 100 mL of sterilized ultrapure water, then frozen in −20 °C and thawed in 37 °C-water bath repeatedly 3–4 times to help proteins released. The protein content was measured by BCA method [39]. The soluble phycobiliprotein (crude extract) was obtained by centrifugation at 12,000× *g* for 20 min, and stored at −20 °C for future use. We could obtain 100 mL of 3.75 mg/mL protein solution from 1 g of *A. platensis* powder and the yield rate was 37.5%.

#### 4.2.2. Preparation of Crude Bacterial Extracellular Protease

The activated seed inoculum was prepared by adding bacterial strains preserved in glycerin into 5 mL 2216E liquid medium (tryptone 5 g/L, yeast extract 1 g/L, Fe_2_(SO_4_)_3_ 0.01 g/L, dissolved in artificial sea water, pH 7.8), and incubating at 16 °C for 16–24 h with 180 rpm shaking. Then, 2% of seed inoculum was transformed to fresh fermentation medium (bean powder 20 g/L, corn powder 20 g/L, wheat bran 10 g/L, Na_2_HPO_4_·12H_2_O 1 g/L, KH_2_PO_4_ 0.3 g/L, CaCl_2_ 1 g/L, Na_2_CO_3_ 1 g/L, dissolved in artificial sea water, pH 7.8) and continuously culturing at the same condition. After 5 days cultivation, the crude enzyme was obtained by collecting the supernatant of the fermentation broth at 12,000× *g* centrifugation for 10 min at 4 °C, and stored at −20 °C prior to use. 

#### 4.2.3. Substrate Immersing Zymography

Substrate immersing zymography was conducted to determine the enzymatic activities against casein according to a previous study, with minor modifications [40]. The SDS-PAGE gel was immersed in the pre-warmed 0.5% (*w/v*) casein solution and incubated at 37 °C for 1 h with 75 rpm shaking. The gel was then stained with 0.1% of Coomassie Brilliant Blue dye solution for 4 h, and then decolored with 30% of ethanol and 70% of acetic acid (*v/v*) until the bands were clearly visible.

### 4.3. Antioxidant Activities of Hydrolysates

#### 4.3.1. Optimization of Hydrolysis Conditions

First, the enzyme/substrate (E: S) ratio (*v*/*w*, mL/mg) was designed as 1:6, 1:8, 1:10, 1:12 and 1:14 with 1 mg/mL enzyme at 50 °C, to optimize the E/S ratio during the hydrolyzing. Then, the hydrolysis reaction was carried out at 35 to 60 °C, with 5 °C at intervals, to estimate the optimal temperature for hydrolyzing. Finally, hydrolyzing was taken from 1 h to 7 h at hourly intervals at 50 °C to optimize the reaction time. The reaction was terminated by heating at 95 °C for 10 min. The reaction buffer was ultrapure water. The hydrolysate was centrifuged at 12,000× *g* and 4 °C for 20 min and the hydrolysis degree was measured by ninhydrin coloration method [41]. The •NH_2_ in hydrolysate could react with ninhydrin and have absorption peak at 570 nm. The standard curve was determined using L-leucine at a concentration of 0, 1, 1.5, 2, 2.5 and 3 mM.

#### 4.3.2. Purification of Hydrolysates

After digestion, the hydrolysates were added into the upper casing of the Amicon^TM^ Ultra-15 centrifugal filter units and centrifuged at 5000× *g* for 30 min to separate peptides with MWCO (molecular weight cut-off) > 3 kDa (in the upper casing) and MWCO < 3 kDa (in the lower casing). 

The peptides with MWCO < 3 kDa was further fractionated by NGC Scout Plus fast protein liquid chromatography (FPLC) system (Bio-Rad, USA) and a XK16/70 column (1.6 × 60 cm) equipped with Sephadex LH-20 gel. One mL of sample was injected into the loading loop and the column was continuously eluted with filtered ultrapure water at a flow rate of 0.75 mL/min. The protein absorbance was monitored at 220 nm with a UV detection all the time. Fractions were lyophilized and redissolved in ultrapure water at the same concentration for further assay.

#### 4.3.3. Determination of Antioxidant Activities

To evaluate antioxidant activities of hydrolytes in vitro, DPPH free radical scavenging activity (DPPH RSA) assay, hydroxyl free radical (OH) scavenging activity (OH RSA) assay and oxygen radical absorbance capacity (ORAC) assay were conducted according to previous studies, with minor modifications [42,43,44]. The DPPH and OH radical scavenging rate were calculated.

The ORAC was defined as Trolox equivalents (mmol TE/g sample or mmol TE/mmol sample) according to the area under the curve (AUC) and calculated using the following formula:

ORAC = (AUC_sample_ − AUC_control_)/(AUC_Trolox_ − AUC_control_) × (M_Trolox_/M_sample_) (1)

where AUC_sample_, AUC_control_ and AUC_Trolox_ were the integral areas under the fluorescence decay curve of the sample, PBS and Trolox, and M_Tolox_ and M_sample_ were the concentrations of the Trolox and sample, respectively.

#### 4.3.4. Protective Effects on Oxidative Damage of Plasmid DNA

To evaluate the protective effects of peptides on the oxidative damage of biological macromolecules (proteins and DNA), the protection effect assay was conducted according to a previous method, with minor modifications [15]. An amount of 8 μL of pET-22b DNA, 2 μL of FeSO_4_ (2 mM), 8 μL of sample and 2 μL of 0.1% H_2_O_2_ were mixed in a sterilized tube and incubated at 37 °C for 10 min. For the blank, 12 μL of sterilized water replaced other reagents incubating with the DNA. For the negative control, sample was replaced with sterilized water. For the positive control, the sample was replaced with Vitamin C (200 μg/mL). The reaction solution was then analyzed by 1% agarose gel electrophoresis under 120 V for 30 min. 

#### 4.3.5. Determination of Intracellular ROS Levels and the Activities of Intracellular Oxidative Enzymes 

HUVECs and HaCaT cells were cultured in RPMI-1640 medium with 10% FBS, 100 U/mL penicillin and 100 μg/mL streptomycin (complete medium) at 37 °C in a humidified atmosphere of 5% CO_2_. Cell viability was measured by MTT assay.

The 2′,7′-dichloro-fluorescence diacetate (DCFH-DA) probe can be oxidized by ROS to produce green fluorescent materials which can be observed under fluorescence microscope. An amount of 1 × 10^5^ of cells were plated in a 24-well plate and incubated overnight. For the HUVECs cells, the medium was replaced with RPMI-1640 medium (without FBS instead, added 35 mM of glucose) for 24 h. For the HaCaT cells, the medium was replaced with RPMI-1640 medium (without FBS, but added 1.5 mM of H_2_O_2_) for 4 h. For the blank, cells were treated with RPMI-1640 medium (without FBS). An amount of 10 μM DCFH-DA probe was added to each well and incubated for 1 h. Cells were washed with PBS for 3 times and re-immersed in 500 μL of PBS. Images were taken using an inverted fluorescence microscope (Axio vert. A1, Carl Zeiss, Oberkochen, Germany).

Next, 2 × 10^6^ of cells were plated in a 6-well plate and treated with the same condition. After incubating for 24 h, cells were washed with PBS for 3 times, then incubating with 150 μL of cell lysis buffer for 30 min on ice. The suspension was centrifuged at 12,000× *g* for 10 min at 4 °C. The protein concentration was measured by BCA method. The CAT, SOD and GSH-Px activities were determined according to the manufacturer’s instructions.

#### 4.3.6. Antioxidant Activities Evaluation Using *C. elegans* Model

##### Maintenance of *C. elegans* and Lifespan Assay

N2 was cultured on nematode growth medium (NGM) plates supplied with *E. coli* OP50 as food at 20 °C. The age-synchronized *C. elegans* were prepared using the alkaline hypochlorite method according to Phaniendra et al. [45]. 

Fresh NGM plates were coated with *E. coli* OP50 containing a different concentration of APs (0.2 and 0.5 mg/mL) and incubated at 37 °C for 48 h to prepare the sample plates. Age-synchronized nematodes (L4 stage) were plated in blank plates and sample plates (80 worms on each plate), as Day 0 for lifespan assay. Nematodes on each plate were transferred to a new plate to avoid new-born worms and counted every day until all nematodes were dead. The survival rate was calculated.

##### Determination of ROS Levels and Activities of Antioxidant Enzymes in *C. elegans*

After 3 days of treatment on the sample plates, nematodes were collected into a M9 buffer (added 100 μM DCFH-DA probe) and incubated at 20 °C for 30 min without light. Nematodes were washed with M9 buffer 3 times. For inverted fluorescence microscope observation, nematodes were anesthetized with 1 μL of NaN_3_ (25 mM) and 20 μL of suspension was dropped onto the center of the slide, and 5 μL of antifade mounting medium was added for observation. 

The ROS accumulation was conducted according to previous studies, with minor modifications [46]. The fluorescence intensity was measured at 15 min intervals for 6 h with excitation 485 nm and emission 530 nm at 25 °C.

For intracellular proteins extraction, nematodes were ultrasonicated on ice and the suspension was centrifuged at 12,000× *g* for 10 min at 4 °C. The protein concentration was measured by BCA method. The CAT, SOD and GSH-Px activities and MDA levels in the supernatant were determined according to the manufacturer’s instructions.

##### Oxidative Stress Experiment

After 3 days of treatment, nematodes were transferred onto NGM plates supplied with 10 mM H_2_O_2_ or 10 mM paraquat, and each plate with 80 worms. The number of survival nematodes was counted per 30 min intervals (the H_2_O_2_ plates) and per 12 h intervals (the paraquat plates) until all the nematodes were dead [47]. The survival rate was calculated. 

After 3 days of treatment, nematodes were transferred onto the fresh NGM plates (80 worms on each plate) and incubated at 35 °C for 10 h [48]. Numbers of survival nematodes were counted after the heat shock and the survival rate was calculated.

#### 4.3.7. Identification and Solid Phase Synthesis of APs

The sequence and composition of peptides in the active fractions were analyzed by liquid chromatography-mass spectrometry (LC-MS/MS) in Sangon Biotech Co., Ltd. (Shanghai, China). The LC-MS/MS was developed on an UltiMate 3000 RSLCnano system (Thermo, Waltham, MA, USA) coupled with a Q Exactive Plus HPLC-Mass Spectrometer (Thermal, Riverside County, CA, USA). The chromatographic separation was achieved using a C18 column (3 μm, 120 Å, 100 μm × 20 mm). The analysis was performed on a C18 analytical column (2 μm, 120 Å, 750 μm × 150 mm). Mobile phase A was 3% DMSO, 0.1% formic acid, and 97% H_2_O and mobile phase B was 3% DMSO, 0.1% formic acid, and 97% ACN. The flow rate was set as 300 nL/min. The MS data was processed with MaxQuant (V1.6.2.10) to identify detected peptides in *Arthrospira platensis* proteomics database. Peptide with maximal abundance was synthesized by solid phase synthesis in ChinaPeptides Co., Ltd. (Shanghai, China) according to the sequence information given by LC-MS/MS.

The structure of peptide was predicted by Phyre2 (http://www.sbg.bio.ic.ac.uk/phyre2 (accessed on 20 December 2022)).

### 4.4. Statistical Analysis

All the experiments and analysis were carried out in triplicate. The Normality of experimental data was analyzed by Shapiro–Wilk test. Normal experimental data were analyzed with by Student *t*-test. Non-parametric data were analyzed by Mann–Whitney test. Data were presented as the mean ± standard deviation (SD) and P < 0.05 was considered as a statistically significant difference.

## 5. Conclusions

Algal protein resources are considered to be a huge treasury of bioactive peptides. More and more researchers are attempting to prepare novel bioactive peptides from algal protein through protease hydrolysis. In addition, novel proteases from marine bacteria also possess great potential in antioxidant peptide preparation, due to their high efficiency and low cost. In this study, phycobiliprotein was hydrolyzed by extracellular proteases from *Pseudoalteromonas* sp. JS4-1 to produce antioxidant peptides. A novel antioxidant peptide INSSDVQGKY was separated and identified from the hydrolysates. The hydrolysates and peptide INSSDVQGKY exhibited excellent antioxidant activities both at cellular levels and in *C. elegans*.

## Figures and Tables

**Table 1:** Peptides identified through liquid chromatography mass spectrum (> 1%).

Amino Acid Sequence	Mass (Da)	Source (Position)	Counts (%)
INSSDVQGKY	1109.535	Allophycocyanin beta-subunit [*Arthrospira platensis* qy3] (9–18)	17.83
VGGSVPREY	962.482	Elongation factor G [*Arthrospira platensis*] (531–539)	9.12
SSYPNRPVP	1015.509	Photosystem II lipoprotein Psb27 [*Arthrospira platensis* C1] (102–110)	8.81
GIFDTMNH	933.401	ATP-dependent Clp protease proteolytic subunit [*Arthrospira platensis* C1] (74–81)	6.91
YTPDYTPK	983.460	Ribulose bisphosphate carboxylase large chain [*Arthrospira platensis* C1] (26–33)	3.34
IDPSHGTGF	929.424	Phospho-2-dehydro-3-deoxyheptonate aldolase [*Arthrospira platensis*] (277–285)	3.33
TPEPKPEPKPEPKPEP	1795.936	CAAD domain-containing protein [*Arthrospira platensis* C1] (52–67)	3.25
YEQMPEPKY	1183.522	NAD(P)H-quinone oxidoreductase subunit K [*Arthrospira platensis* C1] (105–113)	3.14
DVDWSDYQKQ	1282.547	Ferredoxin-NADP reductase [*Arthrospira platensis*] (380–389)	2.78
VGGSVPKEY	934.476	Elongation factor G [*Arthrospira platensis* C1] (496–504)	2.76
TDVPANHPY	1012.461	S-layer domain protein [*Arthrospira platensis* C1] (283–291)	2.42
QGTLEKYI	950.507	Putative adenylate cyclase [*Arthrospira platensis* C1] (375–382)	2.40
AGIDEINRT	987.499	Phycocyanin alpha-subunit [*Arthrospira platensis* qy3] (113–121)	2.32
SESPNLILMDIQMP	1586.768	Multi-sensor hybrid histidine kinase [*Arthrospira platensis* C1] (1700–1713)	2.32
APYDESEVVFH	1291.572	Rod linker polypeptide CpcI [*Arthrospira platensis*] (107–117)	2.20
IDTIPTGGK	900.492	50S ribosomal protein L13 [*Arthrospira platensis* C1] (144–152)	1.96
LIAGGTGPM	815.421	Phycocyanin alpha-subunit [*Arthrospira platensis* qy3] (99–107)	1.92
DVDWSDYQK	1154.488	Ferredoxin-NADP reductase [*Arthrospira platensis*] (380–388)	1.90
LPEEPMTGK	1000.490	Transcriptional regulator AbrB family [*Arthrospira platensis* C1] (9–17)	1.80
VINSSDVQGKY	1208.604	Allophycocyanin beta-subunit [*Arthrospira platensis* qy3] (8–18)	1.68
LVTQQPLGGKAQ	1238.698	DNA-directed RNA polymerase subunit beta [*Arthrospira platensis*] (992–1003)	1.52
KELTKKSPNSP	1227.682	NYN domain-containing protein [*Arthrospira platensis*] (398–408)	1.37
IETKEIPVPT	1125.628	Assimilatory sulfite reductase (ferredoxin) [*Arthrospira platensis* C1] (592–601)	1.34
VDYQEQPREY	1325.589	Major membrane protein I [*Arthrospira platensis* C1] (81–90)	1.30
GANYEDEWK	1110.462	Transketolase [*Arthrospira platensis*] (308–316)	1.30

## Data Availability

The data presented in this study are available on request from the corresponding author. The data are not publicly available due to privacy.

## Supplementary Materials

The following supporting information can be downloaded at: https://www.mdpi.com/article/10.3390/md21020133/s1, Figure S1: Total ion chromatography of F2; Figure S2: MS/MS of INSSDVQGKY.
