# Supplementary figures and images for: Novel Antioxidant Peptides Identified from Arthrospira platensis Hydrolysates Prepared by a Marine Bacterium Pseudoalteromonas sp. JS4-1 Extracellular Protease
Source: Mar Drugs. 2023 Feb 20;21(2):133. doi: 10.3390/md21020133 (PMC9966703; doi:10.3390/md21020133)

## Slide 1
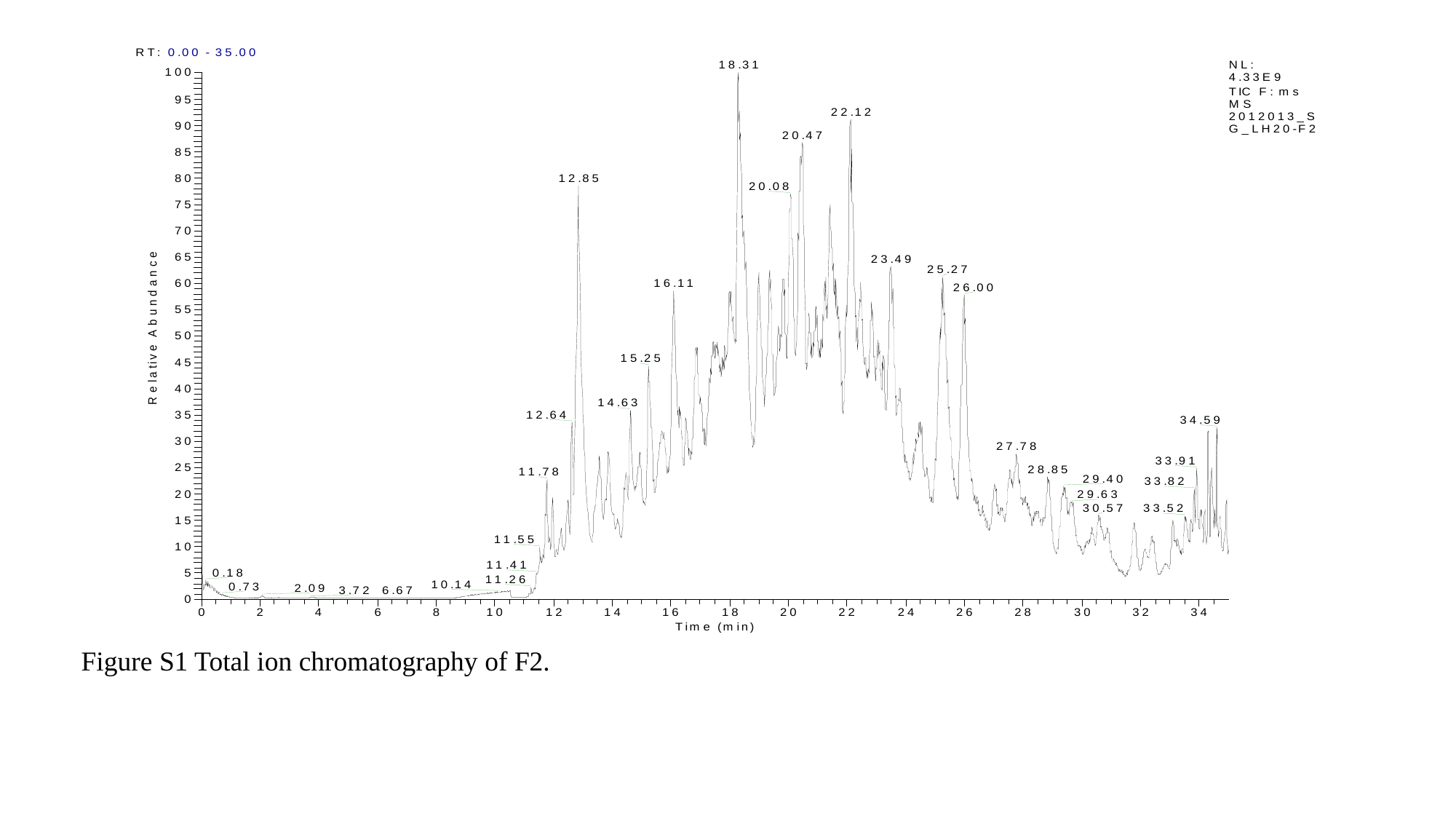

Figure S1 Total ion chromatography of F2.

## Slide 2
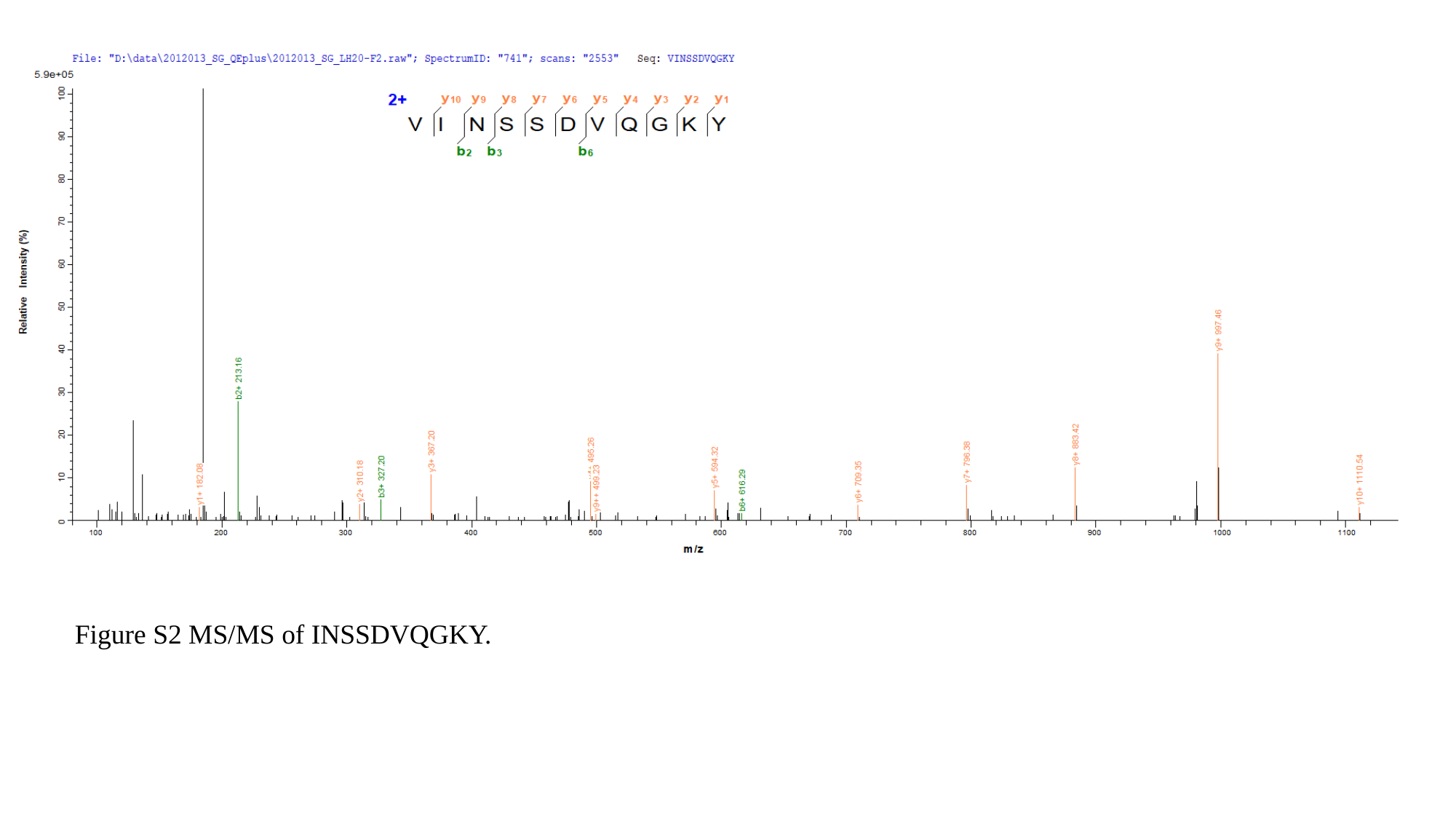

Figure S2 MS/MS of INSSDVQGKY.

Supplement: Supplementary file 1 [file marinedrugs-21-00133-s001.zip › marinedrugs-2163202-supplementary.pptx]
